# Lower limb kinematics improvement after genicular nerve blockade in patients with knee osteoarthritis: a milestone study using inertial sensors

**DOI:** 10.1186/s12891-020-03836-8

**Published:** 2020-12-07

**Authors:** Julien Lebleu, Loic Fonkoue, Eric Bandolo, Herman Fossoh, Philippe Mahaudens, Olivier Cornu, Christine Detrembleur

**Affiliations:** 1 Neuro Musculo Skeletal Lab (NMSK), Institut de Recherche Expérimentale et Clinique, Université catholique de Louvain, Secteur des Sciences de la Santé, Avenue Mounier 53, B-1200 Brussels, Belgium; 2 Faculty of Medicine and Biomedical Sciences, University of Yaoundé 1, PO Box 1364, Yaoundé, Cameroon; 3 Centre Hospitalier Saint Martin De Porres, PO Box 185, Yaoundé, Cameroon; 4 Cliniques Universitaires Saint-Luc, Service d’orthopédie et de traumatologie de l’appareil locomoteur, Avenue Hippocrate 10, B-1200 Brussels, Belgium

**Keywords:** Inertial sensor, Gait, Knee osteoarthritis, Genicular nerve blockade, Biomechanics

## Abstract

**Background:**

Genicular nerve blockade is a possible treatment for patients with knee osteoarthritis. Pain relief and improvement in functioning is expected. This procedure could be of major interest for patients in low-income countries where total knee arthroplasty is not available for the population. This study aims at assessing the immediate benefits on pain, gait, and stairs kinematics after a genicular nerve blockade in patients suffering from knee osteoarthritis in Cameroun.

**Methods:**

A prospective study was carried out on 26 subjects in Cameroun. A genicular nerve blockade was performed on 14 women with painful knee osteoarthritis grade 2–4. Lower limb joint angles were recorded with inertial sensors before and 1 h after injection. Patient-reported outcomes of pain and perceived difficulty were collected, as well as 10 m and 6 min walking tests. A reliability analysis of inertial sensors was performed on a sample of 12 healthy subjects by calculating the intraclass correlation coefficient and the standard error of measurement.

**Results:**

Pain and perceived difficulty decreased significantly (*p* < 0.001). Cadence increased significantly in stairs climbing (upstairs: + 7.7 steps/min; downstairs: + 7.6 steps/min). There was an improvement for hip sagittal range of motion during gait (+ 9.3°) and pelvis transverse range of motion in walking upstairs (− 3.3°). Angular speed range of the knee in the sagittal plane and of the hip in the frontal plane increased significantly in stairs descent (+ 53.7°/s, + 94.5°/s).

**Conclusions:**

This study quantified improvement of gait and stair climbing immediately after a genicular nerve blockade in patients suffering from knee OA in Cameroon. This is the first study objectifying this effect, through wearable sensors.

**Trial registration:**

Pan African Clinical Trial Registry, PACTR202004822698484. Registered 28 March 2020 - Retrospectively registered.

**Supplementary Information:**

The online version contains supplementary material available at 10.1186/s12891-020-03836-8.

## Background

Knee osteoarthritis (OA) affects one third of the population above 65 years [1, 2]. In sub-Saharan Africa, the prevalence may reach up to 33% of the population above 35 years [3]. Pain, local joint swelling, stiffness, and difficulties in the activities of daily living (ADL) are the main symptoms [4].

The goal of knee OA treatment is pain relief and improvement of physical function. One would assume that by using pain-relief therapies, there would almost automatically be improvement in function, but this is not necessarily the case [5]. The lack of a significant correlation between the decrease in perceived pain and the objective improvement of their functional capabilities [6] makes the assessment of the latter essential. Previous studies have assessed the functional quantitative changes of non-surgical pain relief treatment such as oral medications [6, 7], intra-articular injections of hyaluronic acid [8–10], or nonsteroidal anti-inflammatory drugs [11, 12]. They showed positive effects on lower extremity joint kinematics, gait parameters and knee-related functional status on the short term [11, 12], or the long term [8, 9]. However, Shrader et al. underlined that although the relief of knee pain is sufficient to enhance gait function in knee OA, it is insufficient to enhance stair-stepping function [11].

In the past decade, genicular nerve blockade (GNB) and radiofrequency ablation (RFA) appeared as relevant alternatives in the treatment of chronic knee OA pain [13–16]. These procedures are based on the selective inhibition of the consistent sensitive nerves supplying the knee joint capsule, which suppresses the related nerve impulses. This leads to expect a knee pain relief and functional improvement [13, 15, 16]. In comparison to methods that relieve pain by a peripheral action (intra-articular corticosteroids infiltrations, viscosupplementation, etc.) or central (oral analgesics), there is a probable inhibition of proprioceptive impulses. However, only 5 out of the 11 to 13 sensory nerves innervating the joint capsule of the knee are blocked precisely to be effective enough on the pain without completely suppressing proprioceptive influences [17]. These techniques are ambulatory, minimally invasive, with a high potential of pain relief in one single session [13, 18–20]. The average pain relief at 3 months follow-up after a GNB-RFA is 67% improvement from baseline knee pain score, and 95% of these patients still describe pain relief at 6 months [20]. Such procedure could be beneficial for patients in sub-Saharan African settings where total knee arthroplasty (TKA) is difficult to access for the population.

Although previous studies have assessed the effects of GNB and RFA on self-reported measure of pain and function [13, 15, 21–23], little is known about the functional quantitative changes in gait or stair climbing after this treatment. The hypothesis whereby pain relief results in gait improvement should be verified. The studies assessing intra-articular injection were achieved through a motion capture (MoCap) laboratory [8, 11, 12]. This first instrumentation method, a MoCap laboratory, allows for an objective assessment, but is challenging to make use of in low income countries, in particular because of its extensive price, the electrical network and climatic conditions. Another instrumentation method consists of technology based on inertial measurement units (IMU), which are low cost portable electronic devices. They consist of an accelerometer, a gyroscope and often a magnetometer, which enables them to record kinematic data (velocity, acceleration, orientation). Such technology is therefore an opportunity to answer specific research questions in resource-limited settings. Although those sensors have an acceptable validity in comparison to MoCap laboratory [24], the discriminative capacity to detect differences after treatment in population with knee OA has rarely been studied, in particular in out-lab settings [25].

To our knowledge, no study has assessed the quantitative improvement of locomotion after a GNB, especially using inertial sensors. We hypothesized that wearable sensors could detect the quantitative functional effects of peri-articular injection of genicular nerve on gait and stair climbing in patients suffering from knee OA in Cameroun. The aim of this therapeutic pilot study was to assess the ability of inertial sensors to detect differences in kinematics of gait and stairs climbing after a GNB in patients suffering from knee OA, and secondly to assess the immediate benefits on pain, gait, and stairs kinematics after a GNB.

## Methods

### Study design

This interventional study was conducted from September to November 2019 at Centre Hospitalier Dominicain Saint Martin de Porres in Yaoundé, Cameroun. The Central Region Ethics Committee for Human Health Research (Yaoundé, Cameroon) approved the study protocol (agreement number: CE 0–771/CRERSHC/2019) and each patient provided written informed consent prior inclusion in this study.

### Participants

A convenience sample of 26 adults participated in this pilot study. Consecutive patients who presented themselves at the investigators’ consultation within the study period with painful knee OA, who did not respond to conservative therapy, were considered for the study. Radiographic confirmation of knee OA (Kellgren-Lawrence) by a radiologist was required. Patients were included if they suffered from knee pain (Numeric rating scale (NRS) > 5/10) for more than 3 months, not relieved by conservative treatment (oral medication, intra-articular injections with corticoids and viscosupplementation), with a radiological confirmation of tibio-femoral OA grade 2 to 4. Exclusion criteria included other connective tissue diseases that affected the knee, skin lesion on the knee, steroid or hyaluronic acid injection therapy during the previous 3 months, knee surgery scheduled in the next 3 months, anticoagulant medication use, unbalanced diabetes mellitus or hypertension and patients unable to walk. Fourteen adults were recruited to participate in the interventional procedure and 12 healthy adults were recruited by an advertising poster for a reliability assessment (Table 1).

**Table 1:** Characteristics of participants

	Knee OA	Healthy	t-test
	Mean (SD)		***p***-value
**Gender (F/M)**	14/0	7/5	–
**Age (years)**	64.5 (11.3)	50.6 (11.9)	0.787
**Height (m)**	1.61 (0.05)	1.69 (0.07)	0.010
**Weight (kg)**	87.4 (17.2)	71.5 (10.1)	0.184
**Disease start (months)**	50.1 (41.2)	–	–
**Bilateral pain (n)**	7		
	**Median [25–75]**		
**Kellgren-Lawrence**	3 [2–4]	–	

*OA* Osteoarthritis, *SD* Standard deviation, *F* Female, *M* Male, *n* Number of subjects, *[25–75]* Interquartile range

### Experimental protocol

#### Interventional procedure

**Fig. 1:**
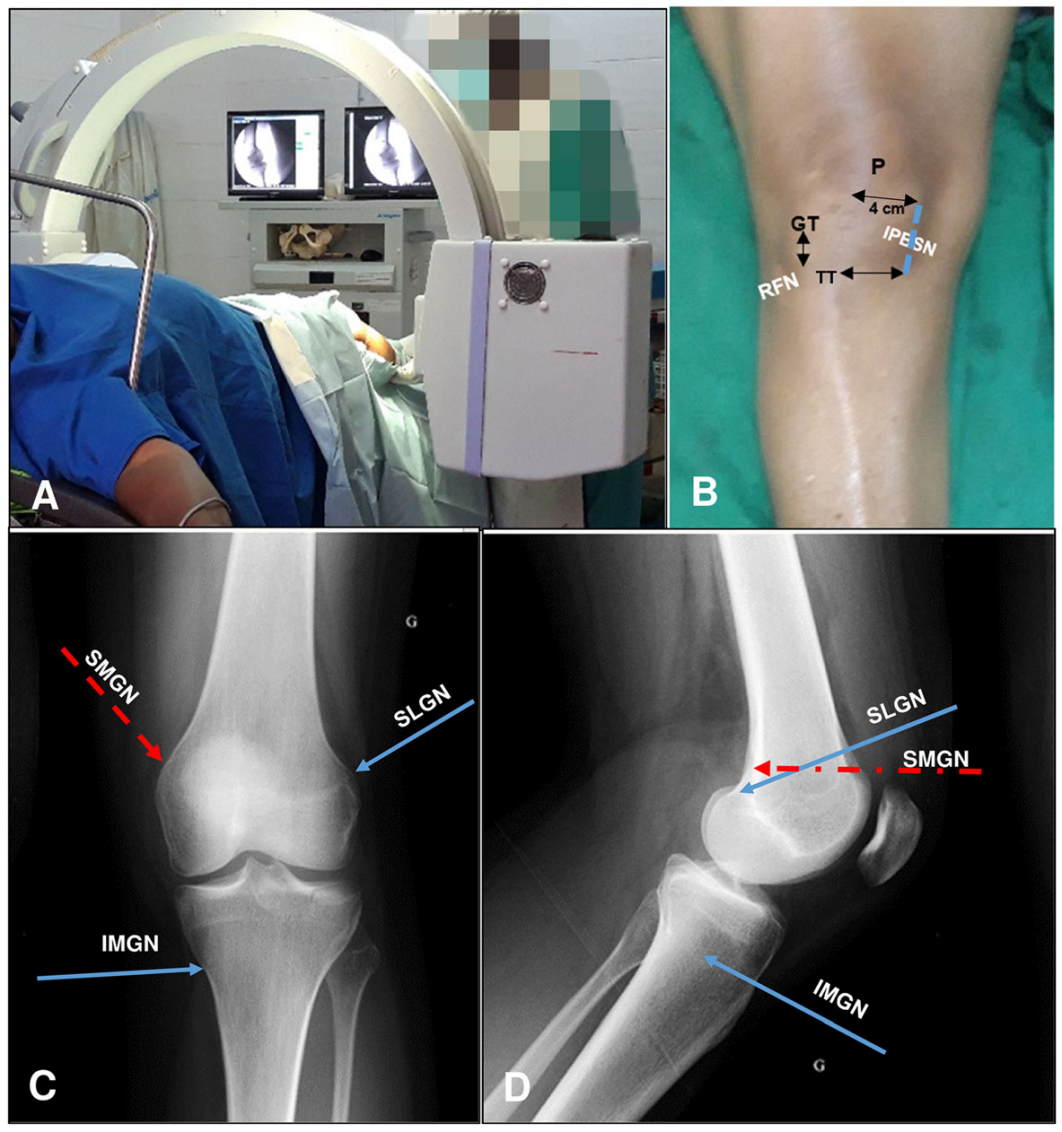
Anatomical targets for fluoroscopic guided genicular nerve blockade. **a** Installation **b** Landmarks for infrapatellar branch of the saphenous nerve (Dashed blue line represents the treatment line) and recurrent fibular nerve (blue point) targeting. **c** Anterior-Posterior X-ray view of the knee. Landmarks of Cannula placement for targeting the Superior medial genicular nerve (Dashed red arrow), superior lateral genicular nerve (Upper blue arrow) and inferior medial genicular nerve (lower blue arrow). **d** Landmarks for targeting the same three nerves on the lateral view of the knee. SLGN, superior lateral genicular nerve; SMGN, superior medial genicular nerve; IMGN, inferior medial genicular nerve; IPBSN, infrapatellar branch of saphenous nerve; RFN, recurrent fibular nerve; P, patella; TT, tibial tuberosity; GT Gerdy’s tubercle

A single treatment session was performed for each of the 14 patients. In case where the patient displayed bilateral knee pains, both knees were treated. The patient was placed in a supine position with a pillow under the popliteal fossa. No premedication or sedatives were administered. Under sterile conditions, the GNB with updated targets [17, 19] was performed with fluoroscopic guidance. At each injection site, skin and soft tissues were anesthetized with 1 mL 1% lidocaine. The five nerves were targeted as referenced above (Fig. 1) [17, 19].

For the superior-lateral genicular nerve (SLGN), a 10 cm 22-gauge radiofrequency (RF) cannula was advanced percutaneously towards the superior edge of the lateral femoral condyle until the tip touched the bone on the anterior posterior (A-P) view. Then the C-arm was rotated to have a true lateral view, with both condyles superimposed. The needle tip was adjusted to fit the target area located at the junction between the superior edge of the lateral condyle and the posterior femoral cortex.

For the superior-medial genicular nerve (SMGN), the RF cannula was advanced towards the superior edge of the medial condyle until the tip touched the bone on AP view. Subsequently, on the lateral view, the tip of the cannula was adjusted to fit in front or just above the adductor tubercle.

For the inferior-medial genicular nerve (IMGN), the RF cannula fitted at the confluence of the medial tibial shaft and the tibial flare in the A-P view, and the midpoint of the tibia in the lateral view.

The recurrent fibular nerve (RFN) was targeted on a longitudinal line drawn below the Gerdy’s tubercle (GT), at a point located 1 cm below the inferior edge of the GT. The RF cannula was inserted at that point and advanced until the tip touched the bone.

For the infrapatellar branch of the saphenous nerve (IPBSN), the treatment target was the longitudinal line connecting both following transversal lines, 4 cm medially to the patellae apex: the transversal line passing by the patellae apex and the one passing by the top of tibial tuberosity. The RF cannula was inserted longitudinally at the proximal edge of the treatment line and advanced deeply in the subcutaneous tissue until the distal edge of the treatment line.

For each of the 5 targeted nerves, after verification of the correct needle placement, a total of 1 mL of lidocaine plus 20 mg of triamcinolone was injected.

#### Assessment

Assessment of patients was performed 1 h before, and 1 h after the interventional procedure by an independent evaluator (JL). All the participants attended the hospital center for the primary data collection session. They were asked to complete the Patient-reported outcome measures (PROM) and their pain intensity after the testing protocol using Numeric Rating Scale (NRS).

The testing protocol included 5 locomotor tasks. The tasks were demonstrated by the operator and were performed in the same order:

(A) walking ten meters at self-selected speed;
(B) walking ten meters at higher speed;
(C) ascend stairs;
(D) descend stairs;
(E) walking freely for 6 min.

After the assessment, their perceived difficulty during the 5 test tasks was assessed on a NRS scale (0–10). Afterwards, participants went to the surgery room for the GNB of the painful knee(s). The same testing protocol was performed 1 h after the intervention. Pain intensity was assessed individually in the cases where the infiltration was performed in both knees.

The same assessment was performed two times in 12 healthy adults. The sensors were removed between the consecutive sessions.

### Equipment

The time to perform 10 m was measured with a standard chronometer. The distance covered during the six-minute walking (E) was assessed with a pedometer (GEONAUTE ONWALK).

To assess the lower limb joint kinematics, seven wearable IMUs; (x-IMU, x-io Techologies, UK) were attached by means of a semi-elastic belt to seven body parts: the waistline at the level of the fifth lumbar vertebra (L5), the middle of the thighs, the middle of the shanks, and at the dorsal side of the feet [24]. Each IMU included a tri-axial accelerometer (full scale ±6 g), a gyroscope (±2000°/s) and a magnetometer (±8.1G) that recorded at sampling frequency of 128 HZ. The IMUs were connected to a computer by means of a Bluetooth connection. Custom application based on open source software was used to record the IMU data (C# program, github.com/xioTechnologies).

### Outcomes

#### Patient-reported outcome measure (PROM)

Pain intensity was measured by a Numeric rating scale (NRS). Function was assessed by the Knee Osteoarthritis Outcome Score (KOOS) [26]. As pain and perceived difficulty are different constructs [27, 28], perceived difficulty during movement was assessed by another NRS scale (0–10).

#### Objective functional assessment of locomotion

##### Clinical outcome

Walking speed (m/s) was assessed twice on a 10 m-track, once at self-selected speed, and once at higher speed. Walking endurance was assessed during a 6 min free walking test. Results are expressed in distance (m).

##### Inertial sensors – kinematics outcome

Each task was segmented with semi-automatic threshold methods based on accelerometer signals (flat zone detection, and peak detection) [29]. Three gait cycles were normalised on 0–100 points and averaged. A cycle lasts from the time point a foot touches the ground until the next contact of the same foot. A combination of vertical shank acceleration and hip and knee angular movement were used to detect those events [29, 30].

The parameters were cadence (step/s), stride time (s), joint range of motion (ROM in degree) and angular speed range (SPEED in °/s). Joint angles of both legs were calculated by a validated method, using the walking functional sensor-to-segment calibration [24]. Three-dimensional joint kinematics of the pelvis, hip, knee, and ankle were calculated based on the recommendation of the international society of biomechanics [6]. Instantaneous three-dimensional angular velocity was calculated by the finite derivative. ROM and SPEED were computed as the difference between the maximum and the minimum in the average gait cycle.

### Statistical analysis

Differences between pre-injection and post-injection conditions were performed with the two tailed paired t-test. The Wilcoxon signed rank test was used for PROM and for variables that failed the test for normality. Statistical significance was defined as *p* < 0.05.

Reliability of the clinical and kinematics outcome was performed on the healthy subjects according to a method described by Wagner [31] using the intraclass correlation coefficient (ICC) and the standard error of the measurement (SEM). ICC consistency parameters were calculated in a 2-way mixed model. SEMs estimate the non-systematic variance. As a measure of within-subject variability among repeated trials, the SEM expresses the measurement error in the same units as those of the original measurement.

Statistical analysis was performed using SPSS (version 25, IMB Corporation, Amonk, NY, USA).

## Results

### Patient reported outcome measure

The score (median [25–75 interquartile range]) for KOOS subscale was 54 [39–72] for symptoms, 56 [34–68] for pain, 47 [44–63] for activities of daily living, and 44 [33–74] for quality of life.

**Fig. 2:**
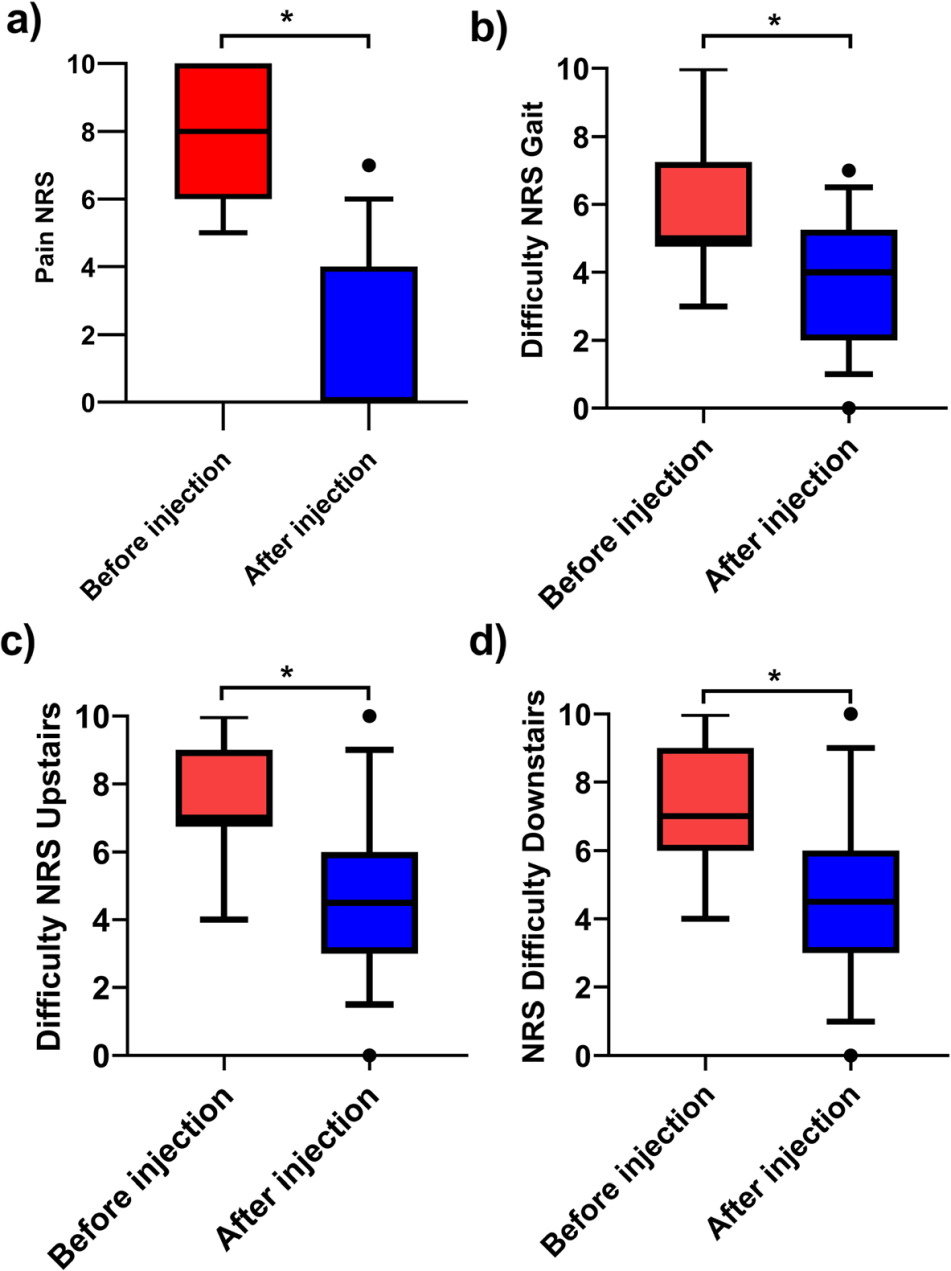
Patient reported outcome during functional tasks: **a** Pain reported on the NRS (most affected knee), **b** Perceived difficulty during gait, **c** Perceived difficulty during ascending upstairs, **d** Perceived difficulty during descending downstairs, * indicate a significant difference (*p* < 0.001)

The pain decreased significantly after the intervention (NRS Pain median [25–75 interquartile range] respectively before and after injection: 8 [6–10], 0 [0–4]) (Fig. 2). The perceived difficulty during the functional tasks also decreased significantly (NRS Gait median [25–75 interquartile range] respectively before and after injection: 5 [4.75; 7.25], 4 [2–5.25], NRS Upstairs before and after injection: 7 [6.75–9], 4.5 [3–6], NRS Downstairs before and after injection: 7 [6–9], 4.5 [3–6]).

### Objective functional assessment of locomotion

#### Clinical outcome

The impact of the intervention on quantitative parameters of locomotion is shown in Table 2. Self-selected walking speed and high walking speed increased significantly after the intervention (mean difference of 0.15 m/s (SD: 0.14) and 0.17 m/s (SD: 0.13) respectively). Walking endurance performance measured by the 6-min free-walking test improved significantly (mean difference of 58 m).

**Table 2:** Clinical and inertial sensor ROM results in functional activities before and after genicular blockade

	Pre	Post	Paired t-test		Reliability	Healthy group
	Mean (SD)		***p*** value	diff	SEM	Mean (SD)
6 min walk						
Distance (m)	286 (137)	319 (127)	**0.037**	58	36	530 (139)
Walking						
High speed (m/s)	1.07 (0.35)	1.19 (0.35)	**0.004**	0.17	0.12	1.45 (0.33)
Walking						
Self-selected speed (m/s)	0.81 (0.27)	0.96 (0.27)	**0.005**	0.15	0.11	1.19 (0.29)
Stride time (s)	1.20 (0.14)	1.15 (0.07)	0.248	−0.05	0.03	1.15 (0.69)
Cadence (step/min)	50.7 (5.8)	52.4 (3.4)	0.319	1.7	1.5	60.4 (16.8)
Pelvis ROM (°)						
Sagittal	5.9 (2.8)	6.2 (3.0)	0.484	0.3	1.5	7.1 (2.6)
Frontal	5.3 (2.2)	6.2 (3.2)	0.321	0.9	1.6	7.5 (1.8)
Transverse	7.5 (3.4)	8.4 (3.5)	0.152	0.9	1.9	7.6 (3.2)
Hip ROM (°)						
Sagittal	28.6 (9.3)	37.9 (7.3)	**0.004**	9.3	4.6	20.3 (6.0)
Frontal	13.6 (5.2)	16.6 (9.1)	0.287	2.9	3.3	9.5 (2.5)
Transverse	15.0 (6.5)	18.4 (7.3)	**0.041**	3.5	3.5	15.6 (4.6)
Knee ROM (°)						
Sagittal	47.0 (17.1)	55.8 (6.0)	0.094	8.8	7.1	68.2 (8.6)
Ankle ROM (°)						
Sagittal	27.5 (6.5)	31.6 (6.1)	0.244	4.1	6.2	49.4 (8.6)
Upstairs						
Stride time (s)	1.84 (0.79)	1.45 (0.42)	**< 0.001**	−0.4	0.1	1.26 (0.43)
Cadence (step/min)	36.4 (10.7)	44.1 (9.8)	**< 0.001**	7.7	3.6	51.0 (11.5)
Pelvis ROM (°)						
Sagittal	12.0 (6.9)	9.4 (4.3)	0.063	−2.6	0.7	6.5 (1.7)
Frontal	12.0 (4.8)	11.5 (5.7)	0.646	−0.6	1.6	6.7 (3.2)
Transverse	17.9 (6.1)	14.5 (5.7)	**0.035**	−3.3	2.3	6.9 (3.8)
Hip ROM (°)						
Sagittal	51.7 (9.8)	48.7 (8.7)	0.349	−3.1	2.1	44.1 (8.5)
Frontal	23.7 (7.1)	20.8 (6.4)	0.242	−2.9	4	12.1 (6.1)
Transverse	26.0 (7.8)	21.5 (7.7)	0.074	−4.4	3.8	14.2 (4.2)
Knee ROM (°)						
Sagittal	67.9 (10.4)	69.9 (12.7)	0.638	1.9	2.9	69.7 (8.2)
Ankle ROM (°)						
Sagittal	37.3 (14.4)	27.3 (7.6)	0.08	−10	4	36.7 (16.0)
Downstairs						
Stride time (s)	1.66 (0.91)	1.31 (0.41)	**0.005**	−0.35	0.2	1.15 (0.69)
Cadence (step/min)	41.5 (12.1)	49.1 (12.0)	**0.005**	7.6	5.1	60.4 (16.8)
Pelvis ROM (°)						
Sagittal	9.6 (2.7)	9.1 (4.3)	0.485	−0.5	1.2	7.1 (2.6)
Frontal	10.6 (4.7)	9.4 (4.5)	0.383	−1.3	0.9	7.5 (1.8)
Transverse	17.8 (9.0)	15.6 (7.3)	0.215	−2.2	0.8	7.6 (3.2)
Hip ROM (°)						
Sagittal	26.5 (7.4)	24.5 (7.5)	0.244	−2	3.6	20.3 (6.0)
Frontal	14.2 (5.3)	16.3 (5.7)	0.394	2.1	2.1	9.5 (2.5)
Transverse	26.4 (9.3)	25.3 (10.3)	0.484	−1.2	2.4	15.6 (4.6)
Knee ROM (°)						
Sagittal	61.0 (14.3)	63.6 (16.1)	0.722	2.6	2.7	68.2 (8.6)
Ankle ROM (°)						
Sagittal	52.3 (16.5)	46.3 (12.4)	0.206	−6	2.1	49.4 (8.6)

The data presented are those from the most painful leg

*SD* Standard deviation, *SEM* Standard error of measurement, *paired t test* Difference between pre-injection parameters and post-injection parameters, *ROM* Range of motion

#### Inertial sensors – kinematics outcome

Cadence and stride time during gait were not significantly affected by the injection, whereas cadence and stride time during ascending and descending stairs evolved significantly towards healthy subject grou*p* values (Mean cadence upstairs for OA patients before injection: 36.4 steps/min, after injection: 44.1 steps/min, healthy group: 51 steps/min; mean difference (SD): 7.7 (5.6); Mean cadence downstairs for OA before injection: 41.5 steps/min, after injection: 49.1 steps/min, healthy group: 60.4 steps/min; mean difference (SD): 8.7 (7.7)).

**Fig. 3:**
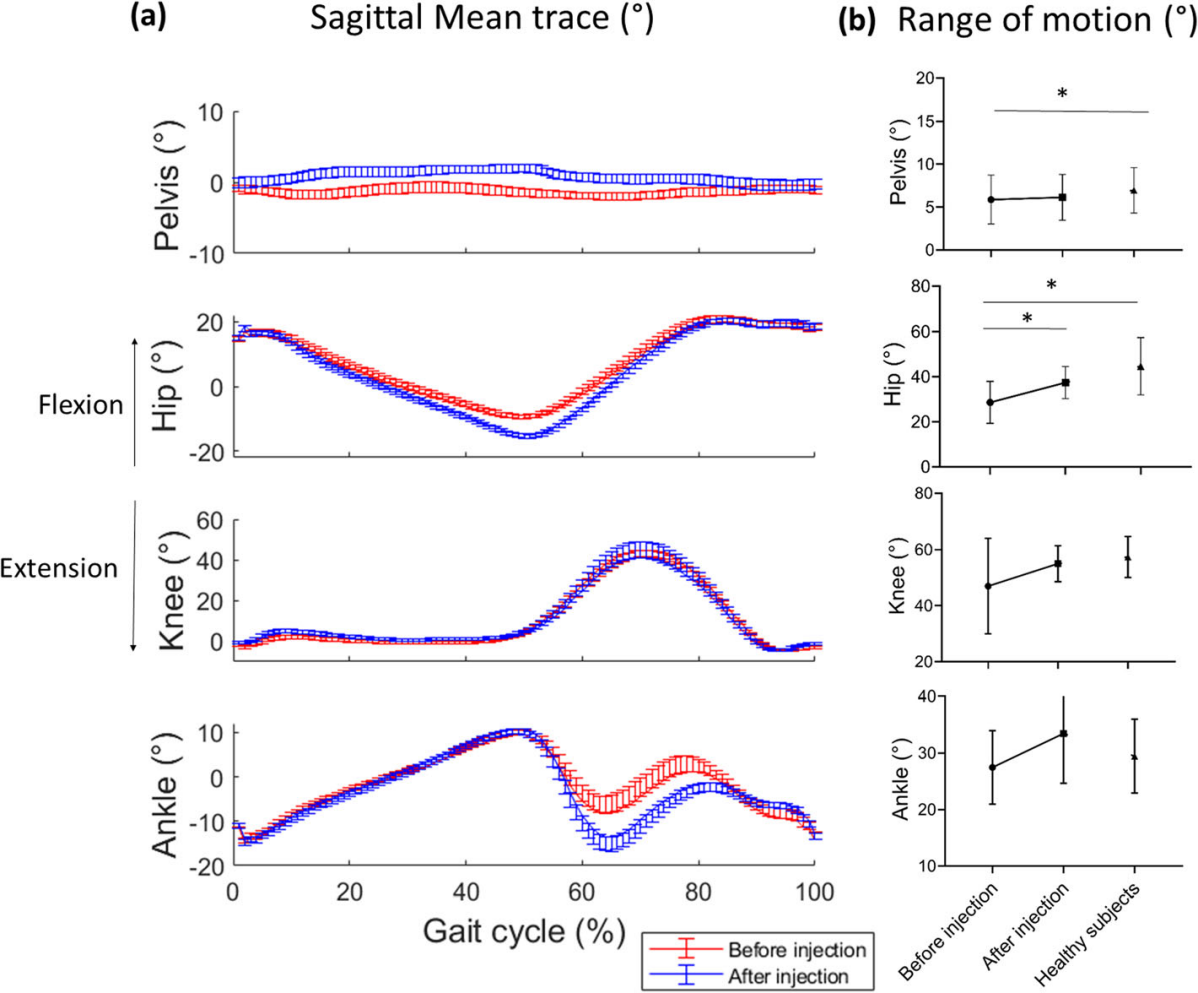
Joint angle in sagittal plane of all subjects for pelvis, hip, knee and ankle during gait: **a** Sagittal mean trace. Error bar display standard error. **b** Range of motion, * indicate significant differences (*p* < 0.05) between post-injection and pre-injection. The black dots on the right represent the healthy subjects group

During gait, sagittal and transverse hip ROM of the most painful side increased significantly of 9.3° and 3.5° respectively. For the hip, the increase in sagittal ROM is manifested by an increase in hip extension at 50% of the gait phase (Fig. 3), while there is a shift in ankle sagittal trace in the swing phase of gait (50–100% of the gait phase). There was no significant difference for knee ROM in the sagittal plane.

For ascending stairs, only pelvis transverse ROM decreased significantly by 3.3°, while no significant ROM differences were observed for descending stairs. Graphs on joints angles for ascending and descending stairs are visible in Additional file 1.

All significant differences were higher than the SEM calculated on the reproducibility test on the healthy subjects.

Angular speed range (SPEED) for the hip and pelvis in walking and ascending stairs increased (Table 3). For descending stairs, hip SPEED in the frontal plane increased significantly by 150% (mean difference of 53.7°/s). Knee SPEED in the sagittal plane also increased significantly by 123% (mean difference 94.5°/s).

**Table 3:** Inertial sensor SPEED results in functional activities before and after genicular blockade

	Pre	Post	Paired t-test		Reliability	Healthy group
	Mean (SD)		***p*** value	diff	SEM	Mean (SD)
Walking						
Pelvis SPEED (°/s)						
Sagittal	59.4 (25.8)	65.7 (29.1)	0.59	6.3	17.4	81.8 (33.6)
Frontal	59.2 (26.7)	71.1 (32.8)	0.133	11.9	8.8	107.9 (38.5)
Transverse	58.7 (17.3)	61.8 (23.8)	0.152	3.1	7.8	89.9 (31.8)
Hip SPEED (°/s)						
Sagittal	191.5 (63.8)	245.7 (46.6)	**0.002**	54.2	29.7	316.3 (85.6)
Frontal	137.0 (55.1)	166.4 (80.6)	0.233	29.4	27.6	131.1 (43.2)
Transverse	180.7 (69.2)	226.4 (105.6)	**0.002**	45.7	34.9	197.4 (61.6)
Knee SPEED (°/s)						
Sagittal	518.5 (179.1)	593.3 (97.4)	0.244	75.1	64.1	655.2 (133.3)
Ankle SPEED (°/s)						
Sagittal	316.8 (129.1)	364.5 (104.8)	0.138	47.7	66	394.5 (106.9)
Upstairs						
Pelvis SPEED (°/s)						
Sagittal	65.2 (27.2)	65.2 (25.2)	0.997	0	7.2	53.6 (14.4)
Frontal	59.3 (22.3)	73.4 (25.5)	0.646	14	19.1	59.5 (26.0)
Transverse	79.8 (22.4)	83.4 (22.0)	**0.035**	3.6	12.9	53.7 (21.8)
Hip SPEED (°/s)						
Sagittal	251.1 (45.2)	278.7 (53.1)	0.349	27.6	12.6	272.2 (40.9)
Frontal	138.5 (48.9)	156.5 (37.8)	0.215	18	21.2	103.7 (50.7)
Transverse	205.5 (73.5)	226.2 (78.8)	0.074	20.6	26.4	158.7 (65.9)
Knee SPEED (°/s)						
Sagittal	436.1 (109.9)	492.6 (81.1)	0.638	56.6	44.2	521.6 (125.5)
Ankle SPEED (°/s)						
Sagittal	354.5 (93.5)	340.3 (101.3)	0.08	−14.2	24.1	362.1 (120.7)
Downstairs						
Pelvis SPEED (°/s)						
Sagittal	70.7 (30.3)	92.9 (40.6)	0.137	22.2	11.1	93.2 (50.8)
Frontal	76.6 (39.0)	83.1 (30.1)	0.542	6.5	15.4	92.2 (37.5)
Transverse	101.0 (33.9)	114.0 (24.9)	0.19	13	16.7	84.7 (27.7)
Hip SPEED (°/s)						
Sagittal	172.7 (55.3)	221.9 (64.4)	0.063	49.2	40.6	245.1 (89.1)
Frontal	109.3 (36.3)	163.0 (63.0)	**0.015**	53.7	15.9	109.2 (39.4)
Transverse	192.2 (85.1)	229.3 (79.4)	0.158	37.1	57.9	219.1 (69.4)
Knee SPEED (°/s)						
Sagittal	407.0 (139.0)	501.4 (93.9)	**0.018**	94.5	37.3	567.9 (145.6)
Ankle SPEED (°/s)						
Sagittal	414.0 (137.7)	462.7 (123.7)	0.232	48.7	39.8	512.4 (117.4)

*SD* Standard deviation, *SEM* Standard error of measurement, *paired t-test* Difference between pre-injection parameters and post-injection parameters, *SPEED* Angular speed range

## Discussion

The most important finding of the present study was that the GNB improved gait kinematics of patients with knee OA immediately. Beyond the subjective improvement in pain and difficulty during the tasks assessed with NRS scale, the assessment using low cost wearable sensors quantified the improvement in gait, ascending and descending stairs.

Although the treatment focused on the knee, the hip ROM increase during gait was the most noteworthy difference observed. We can conclude that the increase in ROM is effectively related to the treatment, as the speed was relatively constant and joint kinematics are speed-dependent during gait [32, 33]. Previous studies have also shown that changes in gait mechanics in the knee joint affect the ROM of the ankle and hip joints [34, 35], possibly explained by the fact that these three joints operate as a kinetic/kinematic chain during gait [34, 36]. It means that problems with one joint are biomechanically related to problems in the others [37, 38]. Skwara et al. obtained similar results combined with an increase in hip and knee ROM after an intra-articular injection [10]. The lack of significant improvement for knee ROM in our sample could be partly explained by broader variability at baseline. On pain therapy, there is no consensus in the literature, as Detrembleur et al. did not find improvement in knee ROM using oral medication [7], while Mehta et al. found significant improvement after intra-articular injection of corticoids and xylocaine [12]. Pain reduction is therefore not always sufficient for improving ROM, which could be explained by the role played by muscle strength in physical functioning [39].

Stair climbing is considered the first affected task in individuals with knee OA [40] with increased hip ROM and decreased knee and ankle ROM. GNB had no impact on this latter kinematic outcome. Similarly to gait, the pain decrease did not result in modification of knee ROM. This means that this treatment is not successful in modifying motor strategy that tends to reduce the ground reaction force moment arm by ambulating with more trunk/hip flexion, less knee flexion, and less ankle dorsiflexion [41]. Asay et al. also found that the degree of severity of OA or pain levels did not seem to affect stair climbing patterns [42]. However, patients with more severe knee OA displayed increased trunk flexion, which was not assessed in this study.

Interestingly the cadence increased and stride time decreased more in stair activities than in gait after the GNB, which means that the overall velocity increased. Moreover, the stride time difference is above the minimal clinically meaningful change of 0.2 s according to Oh Park et al. [43]. This result is consistent with the greater improvement of perceived difficulty during stairs (Fig. 2). We cannot distinguish if the speed increase was mainly in the stance or swing phase of stairs negotiation as we did not record the foot-off events [30]. One can assume that patients lowered their body faster during the stance phase because the subjects increased their joint angular speed at the knee in the sagittal plane and at the hip in the frontal plane when descending stairs [44]. This partially explains the strategies to improve the cadence [45].

Few studies assessed stair kinematics in OA [25], probably due to the complexity of an experimental setup. However, some studies support that there is no particular benefit in measuring more than gait for an indicator of ambulatory functional status [46], our data does not support this statement as the results deliver different insights in motor behaviour. Yet stairs climbing is a high expectation for people receiving treatment for OA [39, 47]. Larger ranges of knee flexion angle and knee flexion moment are required during this task [48, 49] and are therefore more challenging for this population. Furthermore, stair climbing is a single leg activity, where the entire weight of the subject is supported on a single leg and has to withstand the forces of propelling the body upward and forward to the next step [49].

To our knowledge, this is the first study assessing the gait and stairs kinematics after a genicular nerve blockade. It is the second study assessing a clinical population in stairs with inertial sensors [50], and the first one in the African continent. The accessibility of inertial sensors has the potential to increase the clinical understanding of the biomechanics and pathomechanics of the lower limb during daily life activities. It might help surgeons and therapists to integrate scientific findings into clinical examination and management of patients with lower extremity dysfunction [51]. The combined assessment of pain and quantitative, objective outcome of movements appears to be an opportunity to assess the relevant treatments for this population. The inertial sensors allowed to ease the assessment of joint behaviour. In fact, the patients of this study used to wear long dresses that make it difficult to assess movements, even qualitatively. Out of a dedicated laboratory, it is difficult to ask patients to walk half-naked in a hospital. Inertial sensors are helpful in this regard.

Though the final goal of these interventions is to obtain long-lasting improvements, this study examined only the immediate effects of the GNB and found that it was effective in relieving knee OA pain and improving gait kinematics 1 h after the intervention. These results are interesting for pain physicians because the GNB is usually performed as a prelude to the RFA which allows long lasting results. Therefore, assuming that the subsequent radiofrequency ablation would inhibits (by thermocoagulation) the same nerves as the local anaesthetic injected during the prognostic GNB, but for a longer duration, one could envision that the observed benefits last over time. Moreover, the addition of corticoids to the local anaesthetic prolongs the effects of GNB [13, 21], which may be a relevant alternative for the treatment of knee OA pain in poor areas. The results are relevant for physicians in developed countries as well, where GNB and RFA are increasingly performed on patients with intractable knee OA pain who do not qualify for a TKA [14, 16]. Though all the studies assessed the benefits of GNB-RFA on pain and function up to 1 year after the intervention, no previous study assessed the effects on gait kinematics. Further research is expected to assess the duration of improvements found in this study.

The ecological settings of this study are to be highlighted. Although inertial sensors are intended to be used in out-of-lab settings, most of the studies performed in OA population were still conducted in a laboratory environment [25]. The 6 min walking test was adapted with a low-cost sensor to the clinical context, and allowed to easily quantify the improvement in walking long distance. The patients didn’t have to move to a specialised laboratory for the kinematic analysis as the research took place directly in the clinic, using the own buildings’ stairs and corridors. It required few materials and standardization. Although this aspect could be criticized, the low standard error of measurement assessed in the healthy subjects, the same operator for assessment [52], as well as the practical aspect of the study did not diminish the validity of the results. The use of inertial sensors in the GNB seems an added value to represent the whole picture of functional improvement in gait. Clinicians should keep these points in mind when planning, or assessing treatment in knee OA patients. Future studies could assess patients in their everyday environment.

The improvement of motor performance implies more factors than pain alone. The patient’s overall health, the level of strength, abnormalities of the other joints of the lower extremities or spine, or pain avoidance behaviour could be responsible for movement limitations. Eighty percent of our sample presented chronic low back pain at the time of measurement, whereas the other confounding factors were not assessed.

### Limitations

There are some limitations in the current work. First, the exclusive female participant group could be seen as a limitation. However, it is common in Cameroon hospitals that there is a majority female consultation for OA. This could be due to cultural or economic factors. Moreover, the healthy group composed of males and females does not match the OA group.

Second, given the pilot characteristic of this study, data were collected only in the short term and the sample size was low. This limits the clinical applicability and the generalizability of results. The results could only be seen as potential trends. Ongoing work will assess the long-term maintenance of the improvement in a larger population.

## Conclusion

In conclusion, this study showed improvement in hip ROM during gait, and cadence during stair climbing immediately after a genicular nerve blockade in patients suffering from knee OA in Cameroon. Future studies should look at the maintenance of the benefit of GNB and RFA over the longer term. This is the first study objectifying this effect, through wearable sensors. Inertial sensors could be used to detect functional differences after pain relief therapies. This study has the potential to guide clinicians for the choice of injection techniques for OA management.

## Supplementary Information

**Additional file 1.** Joint angle of pelvis, hip, knee and ankle in the sagittal plane during stairs climbing: (a): Ascending stairs. (b) Descending downstairs: Error bar display standard error.

## Data Availability

The data that support the findings of this study are available from the corresponding author upon reasonable request.

## Abbreviations

ADL: Activities of daily living
A-P: Anterior posterior
GNB: Genicular nerve blockade
GT: Gerdy’s tubercle
ICC: Intra-class correlation coefficient
IMU: Inertial measurement unit
IPBSN: Infrapatellar branch of the saphenous nerve
MoCap: Motion capture
NRS: Numeric rating scale
OA: Osteoarthritis
PROM: Patient reported outcome measures
RFA: Radiofrequency ablation
RFN: Recurrent fibular nerve
ROM: Range of motion
SD: Standard deviation
SLGN: Superior-lateral genicular nerve
SMGN: Superior-medial genicular nerve
SPEED: Angular speed range
TKA: Total knee arthroplasty
