# Supplementary figures and images for: Lower limb kinematics improvement after genicular nerve blockade in patients with knee osteoarthritis: a milestone study using inertial sensors
Source: BMC Musculoskelet Disord. 2020 Dec 7;21:822. doi: 10.1186/s12891-020-03836-8 (PMC7722305; doi:10.1186/s12891-020-03836-8)

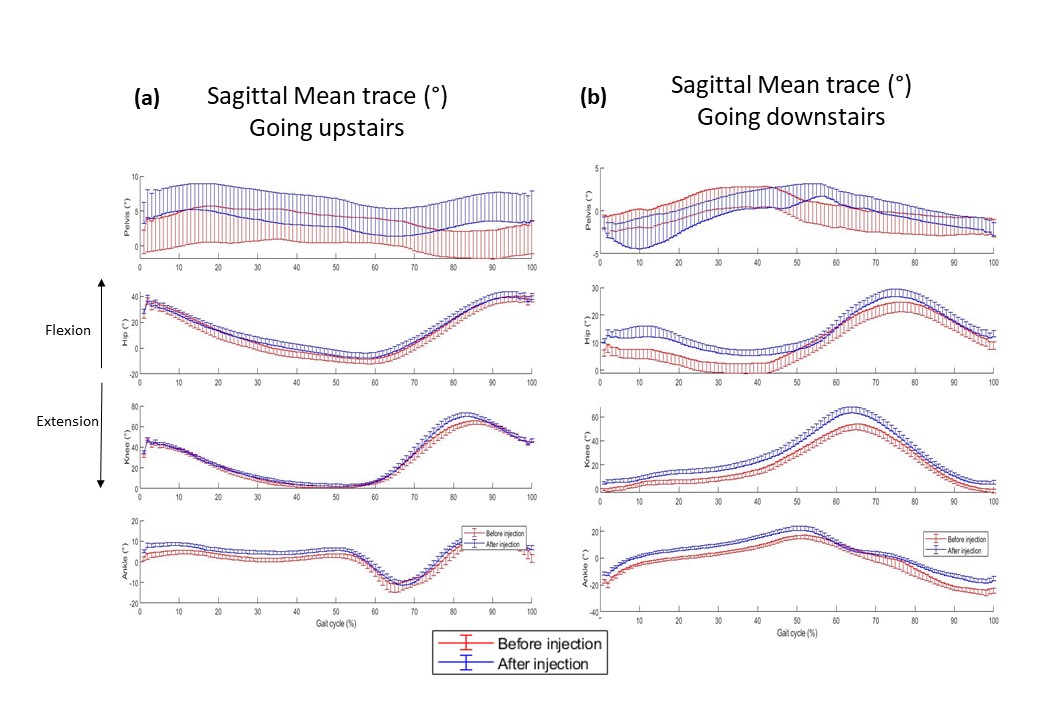

Supplement: Supplementary file 1 — Additional file 1. Joint angle of pelvis, hip, knee and ankle in the sagittal plane during stairs climbing: (a): Ascending stairs. (b) Descending downstairs: Error bar display standard error. [file 12891_2020_3836_MOESM1_ESM.jpg]
